# Deposited pollen from dominant tree genera drives nutrient composition of throughfall in European forests

**DOI:** 10.1007/s10661-026-15883-0

**Published:** 2026-09-09

**Authors:** Elena Gottardini, Fabiana Cristofolini, Antonella Cristofori, Elena Vanguelova, Anne Thimonier, Nicholas Clarke, Laura Wachtveitl, Liisa Ukonmaanaho, Antti-Jussi Lindroos, Manuel Nicolas, Ivan Limić, Pascal Boeckx, Samuel Bodé, Marijn Bauters, Arne Verstraeten

**Affiliations:** 1 Research and Innovation Centre, Fondazione Edmund Mach, San Michele all’Adige (TN), Italy; 2 NBFC, National Biodiversity Future Center, Palermo, Italy; 3 Forest Research, Alice Holt Lodge, Farnham, UK; 4 Swiss Federal Institute for Forest, Snow and Landscape Research (WSL), Birmensdorf, Switzerland; 5 Norwegian Institute of Bioeconomy Research, Ås, Norway; 6 Bavarian State Institute of Forestry, Freising, Germany; 7 Natural Resources Institute Finland (Luke), Helsinki, Finland; 8 Département RDI, Office National Des Forêts, Fontainebleau, France; 9 Institute for Adriatic Crops and Karst Reclamation, Split, Croatia; 10 Q-ForestLab, Department of Environment, Ghent University, Ghent, Belgium; 11 Research Institute for Nature and Forest (INBO), Geraardsbergen, Belgium

**Keywords:** Tree pollen deposition, Atmospheric deposition, Forest nutrient cycle, ICP Forests

## Abstract

Atmospheric deposition is a critical driver of biogeochemical cycling in forest ecosystems. Among the pathways of deposition, throughfall (TF) — precipitation that has interacted with the forest canopy — plays a significant role in the input of atmospheric substances and canopy leachates to the forest floor. We investigated the influence of airborne pollen deposited in TF on its chemical composition across 60 Level II plots of the ICP Forests network in eight European countries. Based on 196 TF samples collected during the 2018 early vegetative season, we identified 53 pollen taxa, with *Pinus*, *Picea*, *Fagus*, and *Quercus* accounting for 91.4% of the total grains. Linear mixed-effects models revealed significant positive links between pollen deposition rates and TF fluxes of several ions, especially potassium (K⁺), which was explained by pollen abundance of all four main tree genera. Additionally, pollen of broadleaved taxa (*Fagus*, *Quercus*) influenced fluxes of the non-purgeable organic carbon (NPOC), dissolved organic nitrogen (DON), ammonium (NH_4_^+^), and phosphate (PO_4_^3−^). Conversely, broadleaved pollen showed a weak negative link with nitrate (NO₃⁻) fluxes, even if its interpretation requires a cautious approach, as several non-mutually exclusive pathways could explain it. Fractionation of the DOC pools highlighted a higher potential for microbial origin in broadleaves than in conifers. These findings provide the first continental-scale evidence of a link between pollen and TF water chemistry, concurrently analyzed on each water sample. This study underscores the ecological significance of pollen beyond plant reproduction and the need to consider flowering phenology and pollen release in forest deposition and nutrient assessments.

**Supplementary Information:**

The online version contains supplementary material available at https://doi.org/10.1007/s10661-026-15883-0.

## Introduction

Substances in the atmosphere can be transferred to forest ecosystems through wet (rain, snow) and dry (gas, particulate) deposition. This process plays a significant role in biogeochemical cycling within forests, influencing soil chemistry, nutrient availability, and overall forest health (Johnson & Lindberg, 1992; McDowell et al., 2020). When passing through the tree canopy, atmospheric precipitation is subjected to changes in chemical composition due to washing and exchange (both leaching and uptake) processes, as shown by the comparison between throughfall (TF) and bulk open-field precipitation samples (Cerniauskas et al., 2024; Grundmann et al., 2024; Limić et al., 2024). The effects of canopy exchange processes have also been confirmed by branch washing and adsorption experiments in *Quercus robur*, *Quercus ilex*, and *Fagus sylvatica* stands (Adriaenssens et al., 2012; Ruiz-Checa et al., 2024).

The canopy exchange processes show seasonal variations that are partly related to the phenological stages of the forest stands (Staelens et al., 2011). In a mixed temperate forest, for example, throughfall was shown to be enriched in ionic nutrient fluxes compared to precipitation, with the enrichment peaking in spring (Grundmann et al., 2024). Likewise, in an oak stand, TF enrichment in potassium (K^+^) peaked in spring and again in autumn (Thimonier et al., 2008). In pine forests, K^+^ deposition was markedly elevated during spring and early summer, which coincided with the phenological phase of flowering (Limić et al., 2024). Similarly, concentrations of base cations (K^+^; calcium, Ca^2+^; magnesium, Mg^2+^; sodium, Na^+^) in TF reached their highest levels in spring in both pine (*Pinus*) and spruce (*Picea*) forests in Finland (Ukonmaanaho et al., 2014). A multi-year study carried out at European level (Verstraeten et al., 2023) highlights the potential role of pollen in precipitation chemistry. The latter study showed that the TF element fluxes measured in different European forest stands were related to seasonal airborne pollen amounts assessed in the surroundings, during the main pollen season.

When flowering, anemophilous tree plants produce — and release into the atmosphere — large amounts of pollen grains, which ultimately deposit on different surfaces (e.g., vegetation, ground, lakes). Pollen amounts collected by an aerobiological sampler may vary significantly within and between years and locations, due to factors such as the distance of the pollen source (Bogawski et al., 2019), plant species composition and abundance (Charalampopoulos et al., 2018), pollen shape and size which influence flying capacity, and meteorological conditions (Pang et al., 2025). For instance, annual *Pinus* and *Picea* pollen deposition, measured as the number of pollen grains deposited per space and time unit, monitored over an 18-year period in ten sites in boreal forest areas of Fennoscandia, ranged from 180 to 2500 (*Pinus*) and from 5 to 300 grains cm^−2^ year^−1^ (*Picea*), depending on the tree species abundance in the surroundings of the sampling sites (Hicks, 2001). Additionally, along a north–south transect in Europe, average annual *Fagus* pollen deposition rate reflected the abundance of beech trees, with values ranging from 1400 grains cm^−2^ year^−1^ in beech-dominated forests to ca. 40 grains cm^−2^ year^−1^ where beech trees were scarce or absent (Pidek et al., 2010).

Pollen contains mono- and oligosaccharides (sugars), proteins, nutrients such as nitrogen (N), potassium (K), sulphur (S), copper (Cu), iron (Fe), zinc (Zn), and other metabolites (Kostić et al., 2015; Valverde et al., 2023). Although the chemical enrichment of TF in comparison to precipitation is well recognised (McDowell et al., 2020), the specific contribution of pollen to TF chemistry has not been directly quantified. Currently, routine throughfall monitoring typically ignores pollen as a variable and its potential role in TF chemistry; consequently, the chemical effects observed in the field may be entirely misattributed only to canopy leaching or atmospheric deposition. With this study, we aim to provide the first continental-scale evidence linking pollen deposition directly to throughfall chemistry using the same water samples.

Specifically, we aim to test the hypothesis that variations in throughfall chemistry in European temperate and boreal forests are driven by pollen abundance during the early vegetative season.

## Methods

### Sampling sites and procedure

**Fig. 1:**
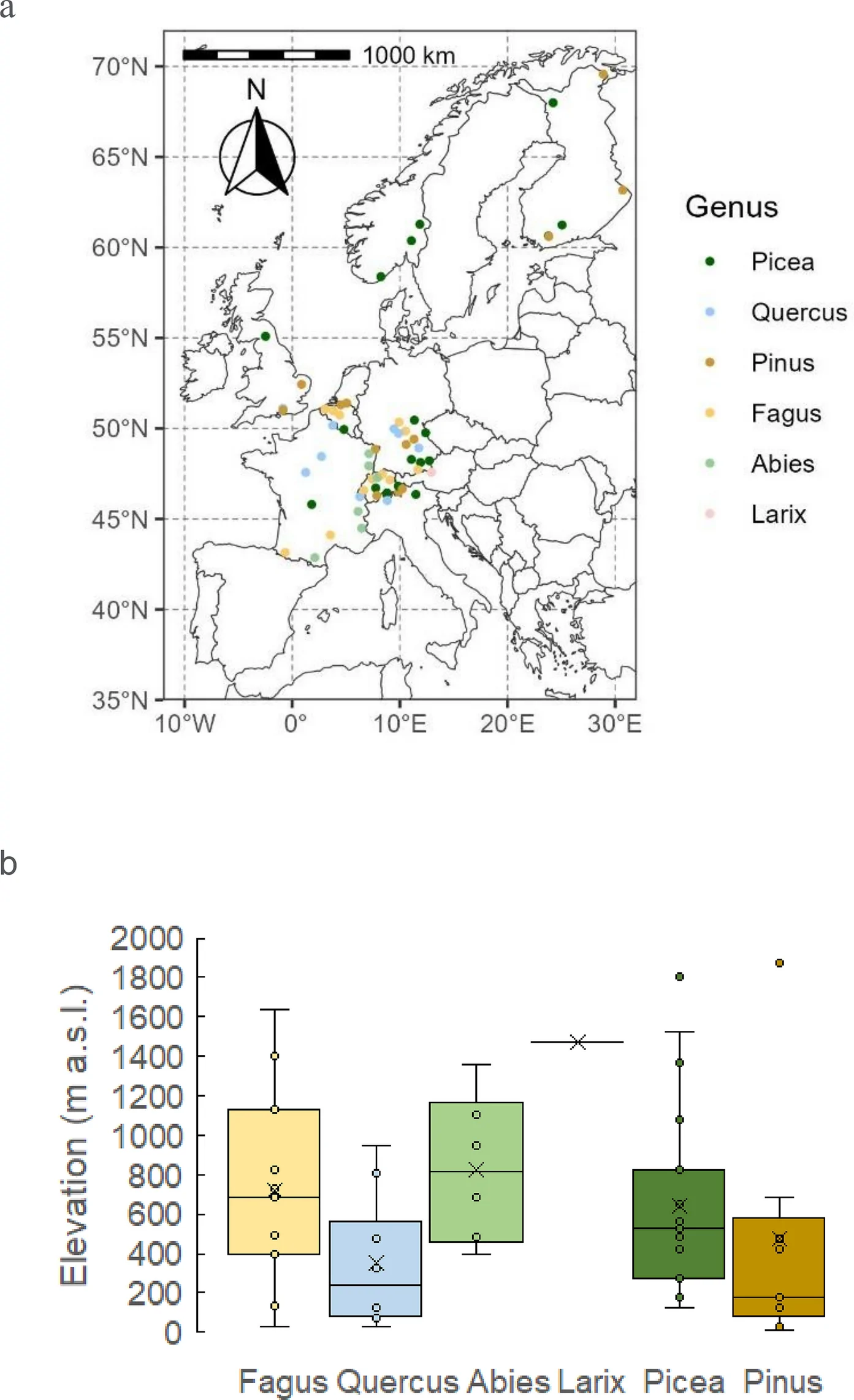
Location of the *n* = 60 ICP Forests intensive monitoring Level II plots where throughfall samples were collected in 2018 **a**, and their altitudinal distribution **b**; each colour represents the dominant tree genus at the plot

The study is based on TF samples collected in 2018 at intensive monitoring plots (Level II) in the frame of the International Co-operative Programme on Assessment and Monitoring of Air Pollution Effects on Forests (ICP Forests; http://icp-forests.net/) (Schwärzel et al., 2025). Generally, a Level II plot has a minimum area of 0.25 ha, is located within a forest area of homogeneous ecological condition and hosts permanent equipment; the plot selection and management, including TF sampling, are within the responsibility of the respective country (Ferretti et al., 2020). TF collectors are generally placed at random positions or according to a systematic grid under the canopy, which means that there is no preference in terms of canopy cover above them (Clarke et al., 2025). For this study, 60 ICP Forests Level II plots across 8 European countries (Belgium, Finland, France, Germany, Italy, Norway, Switzerland and United Kingdom) were considered (Fig. 1a; Table S1). Plots were selected to represent forest stands where the dominant tree genus was *Abies* (*n* = 6 plots), *Fagus* (*n* = 11 plots), *Larix* (*n* = 1 plot), *Picea* (*n* = 19 plots), *Pinus* (*n* = 13 plots), or *Quercus* (*n* = 10 plots). Overall, plots were located along an altitudinal range from 14 to 1900 m a.s.l. (Fig. 1b). A total of *n* = 196 TF samples were collected at the aforementioned Level II plots on a weekly-to-monthly basis and covered, overall, the period from 11th February to 17th July 2018 (Table S1, Fig. S1).

### Pollen analysis

The same TF samples previously analysed for the chemical composition (Verstraeten et al., 2023) were considered for the qualitative and quantitative characterization of pollen deposition. For each TF sample, a water sub-sample (min 15 mL, max 200 mL) was filtered using a 5.0 µm cellulose nitrate mesh filter (Merck Millipore Ltd., Carrigtwohill, Co. Cork, Ireland). After adding a known amount (ca. 155,000) of 10 µm calibrated polystyrene-divinylbenzene** (**DVB) microspheres (SPI Supplies Division Structure Probe, Inc. 206 Garfield Avenue, West Chester, PA 19381–0656 USA), each filter was dissolved in acetone, centrifuged (Eppendorf 5810 R, Hamburg, Germany) at 4000 rpm for 5 min and the supernatant eliminated; the pellet was resuspended with distilled water, centrifuged at 4000 rpm for 5 min and the supernatant eliminated; 3–4 drops of glycerine were added to the pellet, and dried at 65 °C overnight. An aliquot of the pellet (approx. 5–6 drops) was transferred on a slide and embedded in fuchsin jelly for the microscopic pollen identification and quantification. Slides were examined for pollen identification (Faegri & Iversen, 1989); POLIMAGE DB, http://meteo.iasma.it/cgi-bin/pollini/start.cgi?lang=IT&UT=OSPITE&WP=−1, last accessed on 14 April 2025) and counting by highly trained operators. Morphological pollen analysis for taxa identification was performed using an optical microscope (Leitz Diaplan, Ernst Leitz Wetzlar, Wetzlar, Germany) under 400 × magnification. Pollen counts stopped when c.a. 1% of the added microspheres had been counted. Pollen deposition rate (P_d_), i.e. the number of pollen grains (P) per unit of surface area and time (P m^−2^ d^−1^) was then calculated as follows:

1 $${\mathrm{P}}_{\mathrm{d}}\hspace{0.17em}=\hspace{0.17em}((\mathrm{C}\mathrm{P}\times(\mathrm{T}\mathrm{M}/\mathrm{C}\mathrm{M})/\mathrm{V}\mathrm{F}) \times \mathrm{T}\mathrm{Q}) \times \mathrm{t}^{-1}$$

where:

CP = number of counted pollen grains.

TM = number of total microspheres added.

CM = number of counted microspheres.

VF = volume (L) of filtered water.

TQ = TF quantity (L m^−2^).

t = TF sampling length (number of days).

### Throughfall chemical composition analysis

TF sampling and determination of precipitation amount were carried out by the respective countries following the harmonized methods described in the ICP Forests Manual Part XIV (Clarke et al., 2025). After collection, TF samples were stored in the dark at 4 °C and transported to the INBO-laboratory in Belgium. Concentrations of Ca^2+^, K^+^, Mg^2+^, Na^+^, chloride (Cl^−^), phosphate (PO_4_^3−^), sulphate (SO_4_^2−^), nitrate-nitrogen (NO_3_^−^), nitrite-nitrogen (NO_2_^−^) and ammonium-nitrogen (NH_4_^+^) were determined on filtered (0.20 µm syringe filter, Chromafil A20/25) subsamples using a Metrohm IC 930 ion chromatograph (Metrohm AG, Antwerp, Belgium). Concentrations of total dissolved nitrogen (TDN) and non-purgeable organic carbon (NPOC) were measured on unfiltered acidified subsamples with a Formacs^HT^ TOC/TN analyzer (Skalar, Breda, Netherlands) after a preliminary test showed that filtration had no effect on the results of this method. The limits of quantification (LOQ) of solutes are:

0.1 mg L^−1^ for Ca^2+^, Na^+^, Cl^−^, PO_4_^3−^, SO_4_^2−^, NO_3_^−^, and NO_2_^−^; 0.05 mg L^−1^ for K, Mg, and NH_4_^+^; 0.5 mg L^−1^ for TDN; 1 mg L^−1^ for NPOC.

The concentrations of dissolved organic nitrogen (DON) were calculated by subtracting the sum of inorganic nitrogen forms from total dissolved nitrogen TDN as follows:

2 $$\mathrm{D}\mathrm{O}\mathrm{N}\hspace{0.17em}=\hspace{0.17em}\mathrm{T}\mathrm{D}\mathrm{N}-({\mathrm{NO}}_{{3}^{-}}\mathrm{N}\hspace{0.17em}+\hspace{0.17em}{\mathrm{NO}}_{{2}^{-}}\mathrm{N}\hspace{0.17em}+\hspace{0.17em}{\mathrm{NH}}_{{4}^{+}}\mathrm{N})$$

Quality assurance in the ICP Forests programme is ensured through routine reviews of sampling and analysis procedures by the Expert Panels, laboratory ring tests, and data validation processes as outlined in the ICP Forests Manual Part XVI (Fürst et al., 2020).

### DOC fractionation

Dissolved organic carbon (DOC; mg L⁻^1^) was quantified as total organic carbon in TF following filtration. TF subsamples were passed through 0.45 μm cellulose nitrate membrane filters (Fisher Scientific) and analyzed on a TOC analyzer (TOC-L, Shimadzu Corporation, Japan).

Dissolved organic carbon quality was further described using three indices: SUVA₂₅₄ (specific ultraviolet absorbance at 254 nm), the humification index (HIX), and the fluorescence index (FI). The specific ultraviolet absorbance SUVA₂₅₄, used as a proxy for the aromatic and comparatively recalcitrant fraction of DOC (Hansen et al., 2016), was determined from UV absorbance of the filtered samples measured at 254 nm with a spectrophotometer (Jenway 5000, Jenway, UK). Ultrapure water served as the baseline, and blanks were run after every five samples. SUVA₂₅₄ (L mg⁻^1^ m⁻^1^) was calculated as:

3 $${\mathrm{S}\mathrm{U}\mathrm{V}\mathrm{A}}_{254}\hspace{0.17em}=\hspace{0.17em}(\mathrm{A}\mathrm{b}\mathrm{s}\mathrm{o}\mathrm{r}\mathrm{b}\mathrm{a}\mathrm{n}\mathrm{c}\mathrm{e}254 [{\mathrm{c}\mathrm{m}}^{-1}]/\mathrm{D}\mathrm{O}\mathrm{C} [\mathrm{m}\mathrm{g} \times{\mathrm{L}}^{-1}])\hspace{0.17em}\times \hspace{0.17em}100 [\mathrm{c}\mathrm{m}\ \times {\mathrm{m}}^{-1}]$$

Fluorescence characteristics of DOC were measured with a microplate reader (SpectraMax i3x, Molecular Devices, USA). For HIX, emission spectra were recorded at an excitation wavelength of 254 nm over 300–480 nm in 5 nm increments (Ohno, 2002), and the index was calculated as:

4 $$\mathrm{H}\mathrm{I}\mathrm{X}\hspace{0.17em}=\hspace{0.17em}{\Sigma \mathrm{I}}_{435\to 480}/ ({\Sigma \mathrm{I}}_{300\to 345}\hspace{0.17em}+\hspace{0.17em}{\Sigma \mathrm{I}}_{435\to 480})$$

where *I* is fluorescence intensity.

The humification index HIX reflects the degree of humification, i.e. the extent to which organic matter has been decomposed and transformed into humic substances.

The fluorescence index FI was calculated as the ratio of emission intensity at 470 nm to that at 520 nm at an excitation wavelength of 370 nm (McKnight et al., 2001). The index FI is used to infer DOC source, with values > 1.9 typically indicating predominantly microbial-derived DOC (e.g. bacterial lysates and extracellular products) and values < 1.4 suggesting a stronger contribution from terrestrial plant material, such as lignin-derived compounds (McKnight et al., 2001).

### Data analysis

Pollen deposition rates (i.e., the number of pollen grains deposited per unit of surface area and time) of all the taxa identified in the *n* = 196 TF samples were considered to describe the overall pollen spectrum, i.e., the pollen taxa list and relative abundances. In addition, pollen deposition rate percentages were considered separately at the level of the six stand types defined by the dominant tree genus.

The variations over time of *Fagus*, *Quercus*, *Picea*, and *Pinus* pollen abundance (i.e., the pollen curves) were obtained by averaging all the deposition rate values with the same sampling starting date (i.e., the week number) within the respective taxon; results were min–max normalized for the graphical representation. *Abies* and *Larix* were excluded, also for the following analyses, because TF samples did not cover — thus were not representative for — the flowering period.

By combining the main flowering period of each tree genus, as reported by Bledý et al. (2024) and Meng et al. (2022), and the temporal variation of pollen amounts recorded in this study, the main pollen season (MPS) period was defined for each taxon by including, if necessary, up to three weeks before and after the pollen peak date.

Differences in TF compound fluxes between tree genera were tested with the nonparametric Kruskal–Wallis test (TIBCO Statistica 14.0.1.25), considering the data over the period April–June 2018 (weeks 14–26), common for the four tree genera. Differences in DOC characteristics (SUVA_254_, HIX, FI) between tree genera were tested with the nonparametric kruskalmc multiple comparison test using the R package ‘pgirmess’.

Statistical analysis was performed using R version 4.4.1 (R Core Team, 2024) within the RStudio environment (version 2024.06.1). Linear mixed-effects models (LME) for the TF fluxes as a function of mean weekly pollen deposition rates in the MPS were evaluated for the four genera *Fagus*, *Quercus*, *Picea* and *Pinus*, and the sum of the two broadleaves (*Fagus* + *Quercus*) and the two conifers (*Picea* + *Pinus*), using the R package ‘lme4’ (Bates et al., 2015). Deposition rates (adding + 1 because the data contains zeroes) and TF fluxes were logtransformed to better approach a non-normal distribution. Plot ID was included as a random effect to account for differences in location and sampling methods. A compound symmetry structure corresponding to uniform correlation (corCompSymm) was included to account for autocorrelation between repeated samples at the same location if significant. This resulted in the following model formula (y = chemical variable, i = tree genus, j = Level II plot, ε_ij = residual error):

5 $$\mathrm{l}\mathrm{o}\mathrm{g}(\mathrm{T}\mathrm{F}\mathrm{f}\mathrm{l}\mathrm{u}\mathrm{x}\_\mathrm{y})\hspace{0.17em}\sim \hspace{0.17em}\mathrm{l}\mathrm{o}\mathrm{g}(\mathrm{P}\mathrm{s}\_\mathrm{i})\hspace{0.17em}+\hspace{0.17em}\mathrm{r}\mathrm{a}\mathrm{n}\mathrm{d}\mathrm{o}\mathrm{m}(\mathrm{p}\mathrm{l}\mathrm{o}\mathrm{t}\_\mathrm{j})\hspace{0.17em}+\hspace{0.17em}\mathrm{c}\mathrm{o}\mathrm{r}\mathrm{C}\mathrm{o}\mathrm{m}\mathrm{p}\mathrm{S}\mathrm{y}\mathrm{m}\mathrm{m}(\sim \hspace{0.17em}1|\mathrm{p}\mathrm{l}\mathrm{o}\mathrm{t}\_\mathrm{j})\hspace{0.17em}+\hspace{0.17em}\upvarepsilon \_\mathrm{i}\mathrm{j}$$

The validity of models was checked based on histograms of the residuals and homogeneity of residuals variance in function of the fitted values.

## Results

**Fig. 2:**
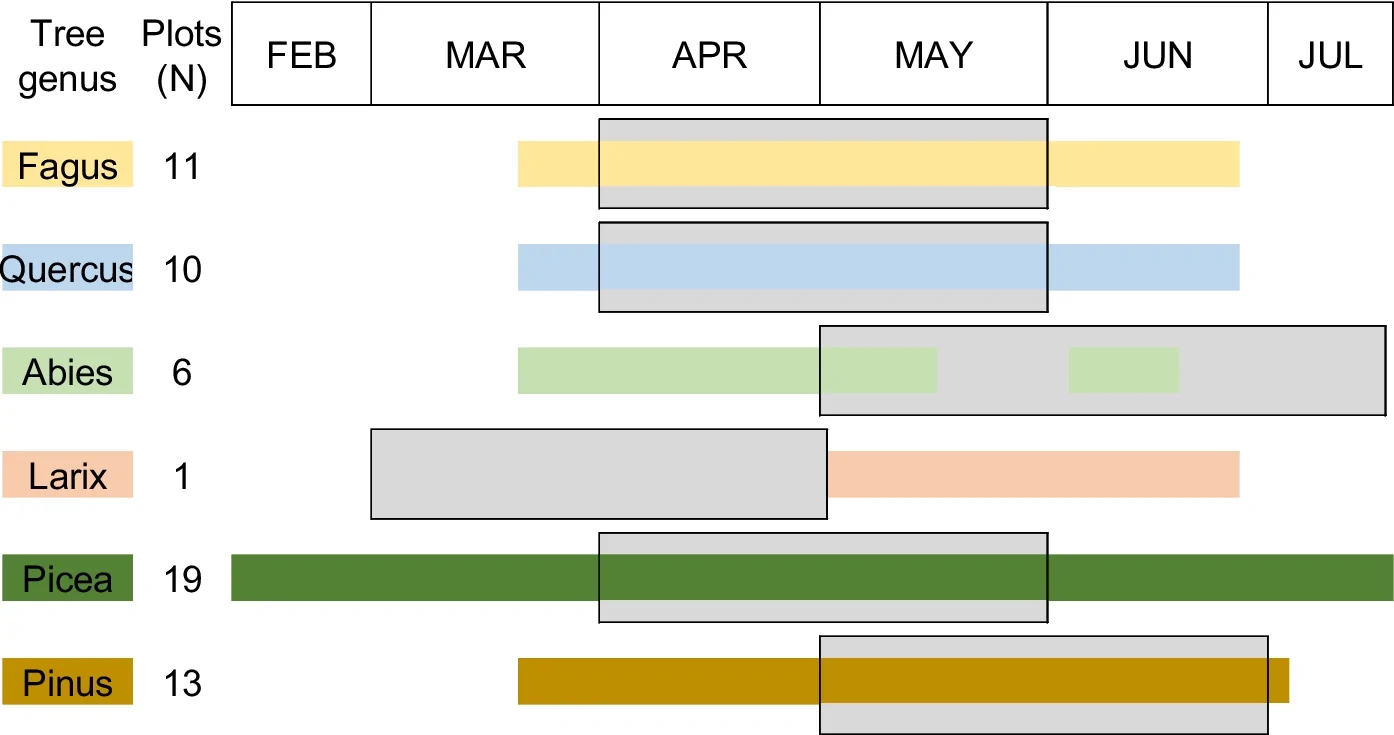
Temporal coverage of TF samples collected in 2018 in *n* = 60 ICP Forests Level II plots across Europe, grouped by the main tree genus on the plot (coloured bars); the flowering months for each tree genus are shown in grey (following Bledý et al., 2024 for *Abies*, and Meng et al., 2022 for the other genera). Timing and length of all the 196 TF samples are reported in the Supplementary Material, Fig. S1

Overall, 57% of TF samples fell within the respective tree genus flowering period for at least 50% of their sampling duration (Fig. 2; Fig. S1); 46% and 100% of TF samples in *Abies* and *Larix* stands were out of the respective flowering periods, thus not being representative for the pollen deposition. As for the sampling frequency of TF samples, the largest proportion (44.9%) corresponded to 1 week; 14.3% to 2 weeks, and 40.8% to 3–5 weeks (Fig. S1).

### Pollen deposition

**Fig. 3:**
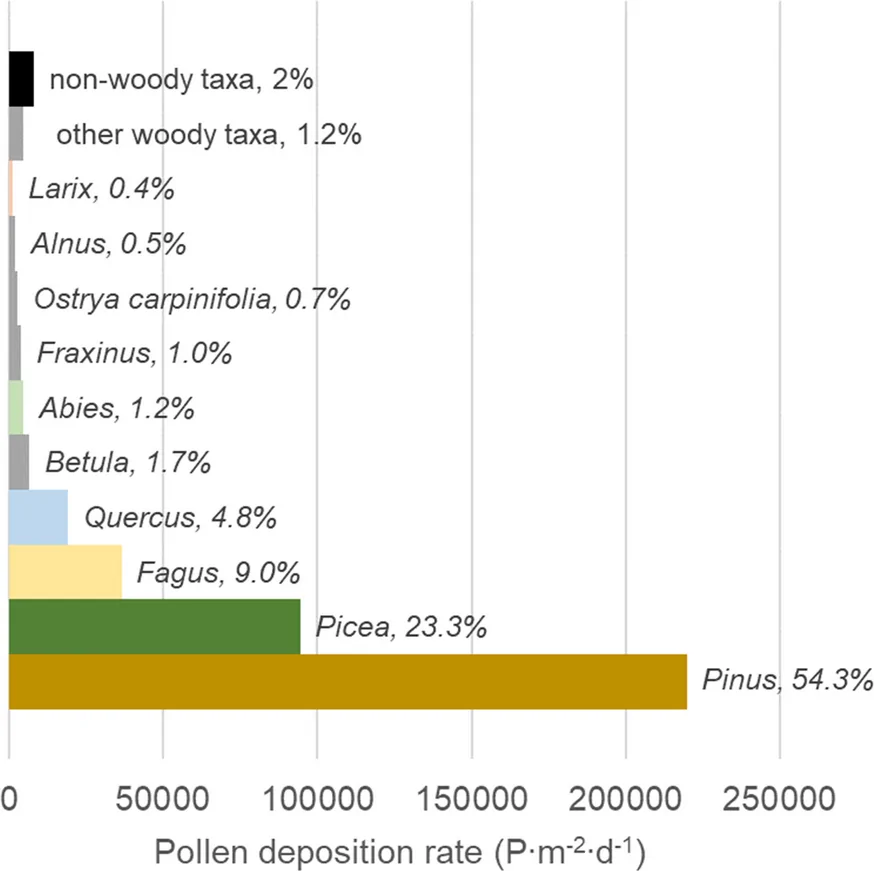
Mean number of pollen grains deposited per unit surface area and time (pollen deposition rate) for each of the ten main woody taxa, the other woody taxa, and the total of non-woody taxa, assessed in the 196 throughfall samples collected in 2018 in 60 ICP Forests Level II plots across Europe, and percentage values in the total pollen

Overall, 53 pollen taxa were identified in the *n* = 196 TF samples, 30 of which belonged to woody species (98% of the total pollen counts), and 23 to non-woody taxa (2% of the total pollen counts). *Pinus* was the most abundant pollen, representing more than half of the total pollen, followed by *Picea*, *Fagus* and *Quercus*, all together representing 91.4% of total pollen (Fig. 3). Among the non-woody taxa, Poaceae pollen was the most abundant (76.6% of the non-woody taxa; 1.6% of the total pollen), followed by Urticaceae (5.1% of the non-woody taxa; 0.1% of the total pollen) and Asteraceae (3.4% of the non-woody taxa).

**Fig. 4:**
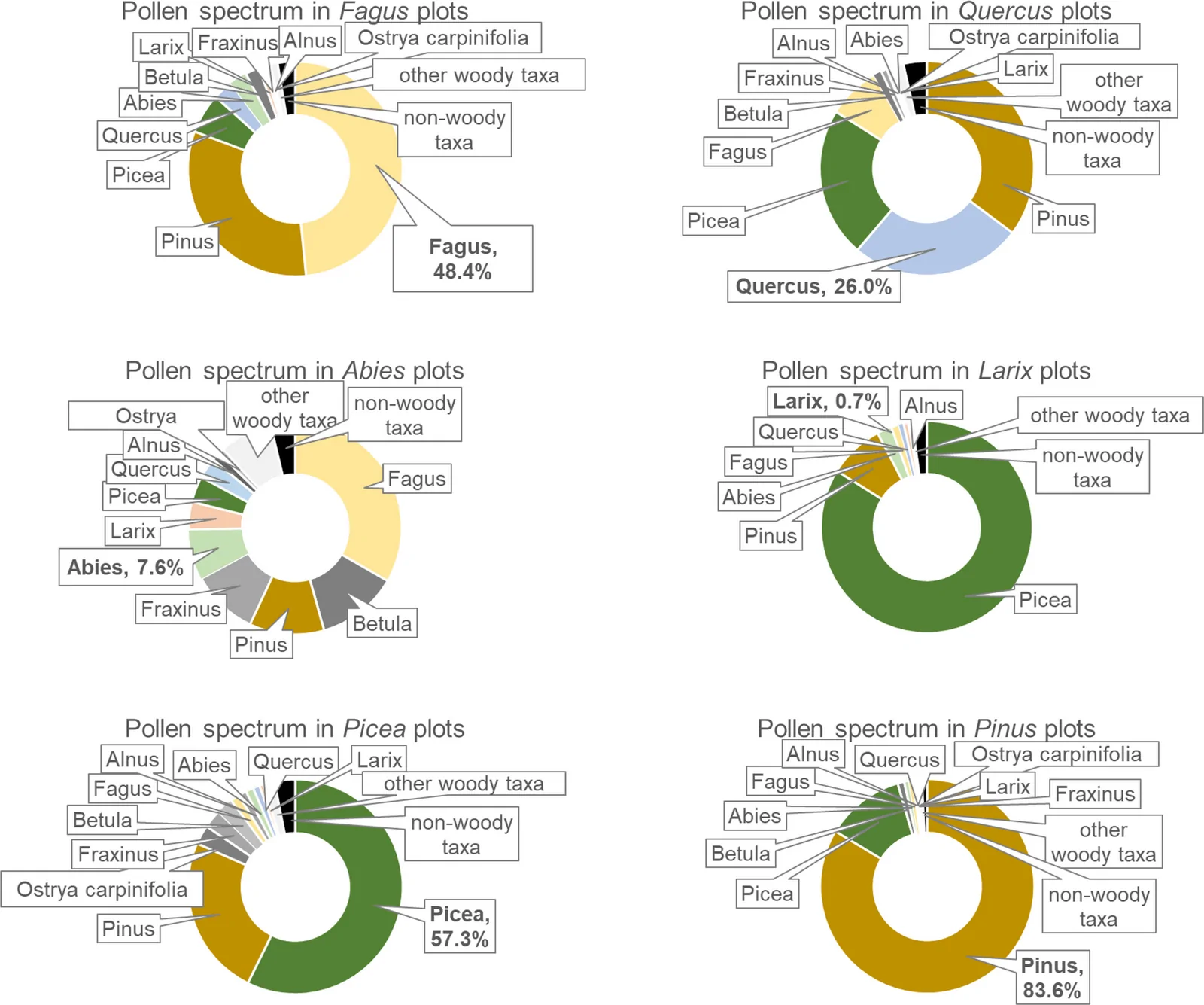
Relative pollen amounts of the ten most abundant woody taxa, the other woody taxa aggregated, and the non-woody taxa (i.e., the pollen spectrum) detected in the 196 throughfall samples collected in 2018 in 60 ICP Forests Level II plots across Europe, for each of the six tree genus stand types; the pollen taxon, with the related percentage value, corresponding to the main tree genus is given in bold

In *Fagus*, *Picea*, and *Pinus* stands, the most abundant pollen detected in the TF samples belongs to the corresponding tree genus (49.7%, 59.1%, 84.3%, respectively); in *Quercus* stands, the corresponding pollen (26.9%) is second in quantity after *Pinus* (Fig. 4). In *Abies* stands, *Fagus* pollen recorded the highest values, likely because the TF sampling happened earlier than the *Abies* flowering phenological phase (see Fig. 3); *Abies* pollen (7.9%) was only the fifth in abundance. In the *Larix* stand (only one plot), the corresponding pollen was scarce (0.7%), while the prevailing taxon was *Picea* (85.8%), likely because the TF sampling took place after the *Larix* flowering phenological phase (see Fig. 2). Examples of the main pollen types detected in the TF samples, and related morphological characteristics examined for their identification are shown in Fig. S2.

**Fig. 5:**
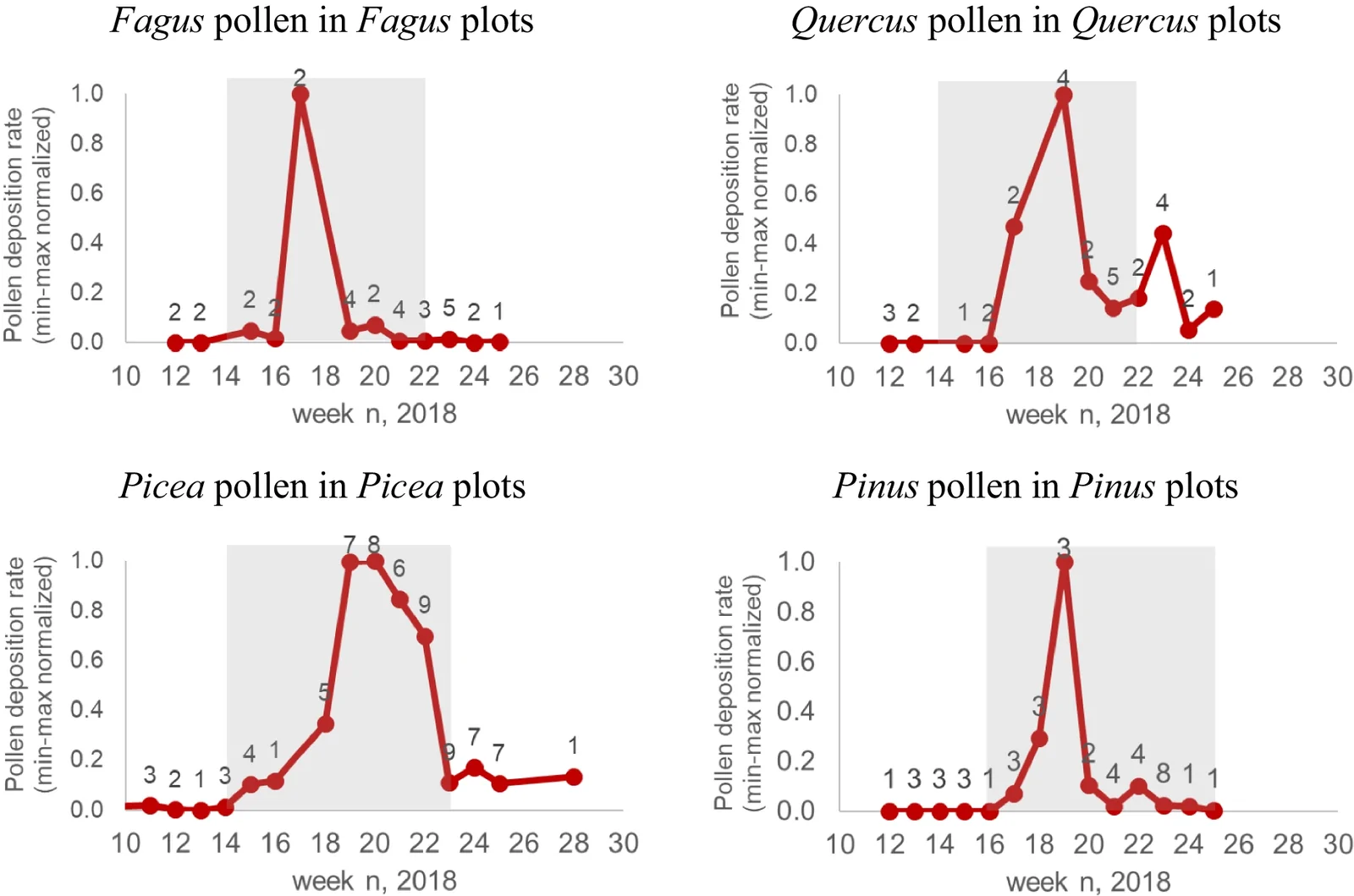
Time courses of the normalized mean deposition rates of *Fagus, Quercus, Picea*, and *Pinus* pollen assessed in the TF samples collected in all stands of the corresponding tree genus. The number of TF values averaged for each week is also reported. Grey areas represent the main pollen seasons (MPS)

The variations over time of the main pollen taxa abundances allowed us to more precisely define the MPSs, evidenced by the grey areas in Fig. 5. Both *Fagus* and *Quercus* MPSs span April–May (weeks 14–22). *Fagus* pollen curve peaks in the last week of April (week 17). *Quercus* displays a first, greater, peak at week 19, and a second smaller one at week 23; this is likely attributable to the presence of different *Quercus* species, flowering at slightly different times, as well as the wide spatial and altitudinal distribution of the plots (Fig. 1; Table S1) that in turn influences flowering phenology of the same species. *Picea* MPS covers the weeks 14–23, peaking around weeks 19–20. *Pinus* MPS covers weeks 16–25 and shows the maximum value at week 19; a second small peak is evidenced at week 22, probably due to the presence of different species and different lati-altitudinal distributions, as for *Quercus*.

### Throughfall compound fluxes

**Fig. 6:**
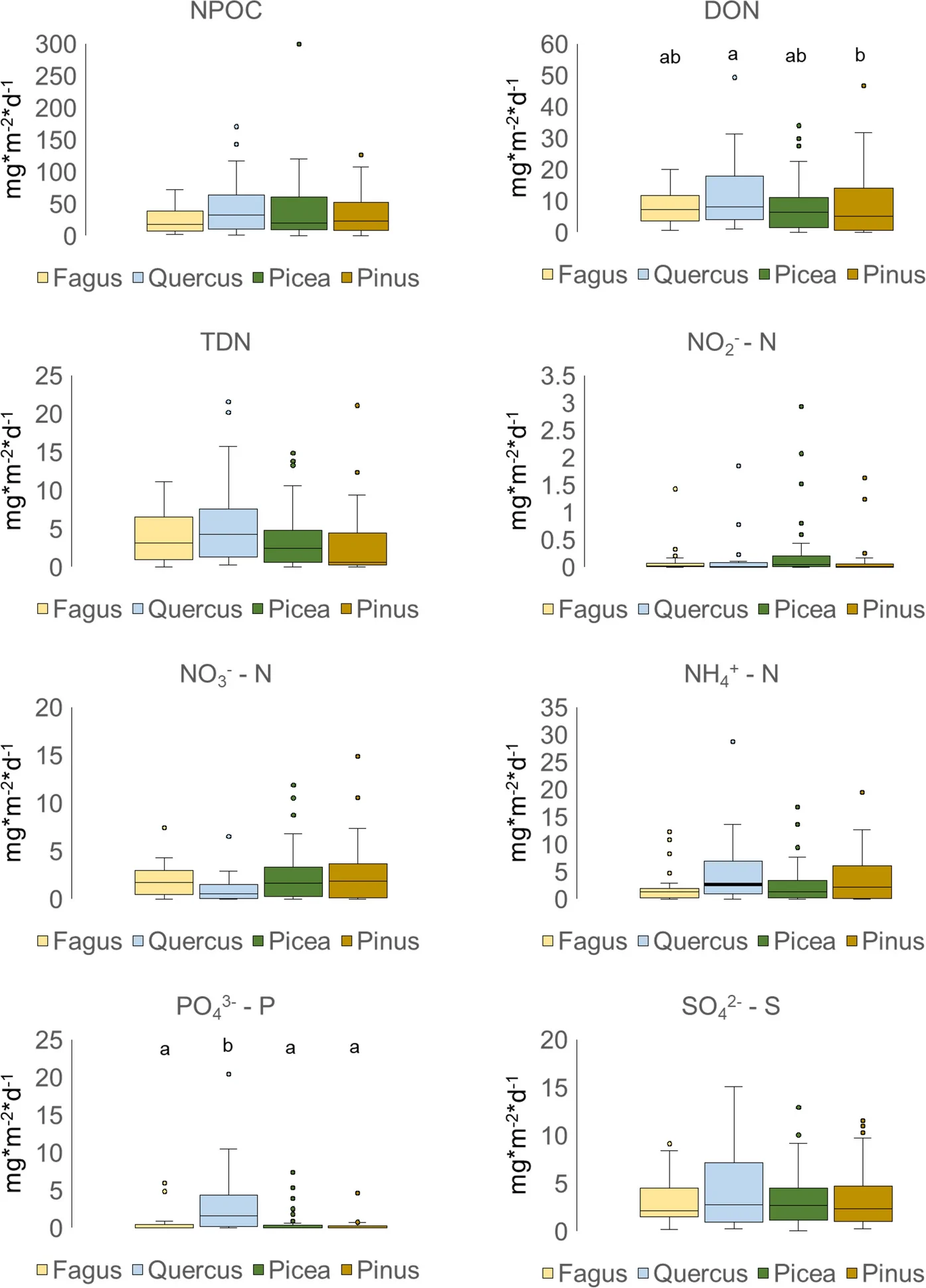

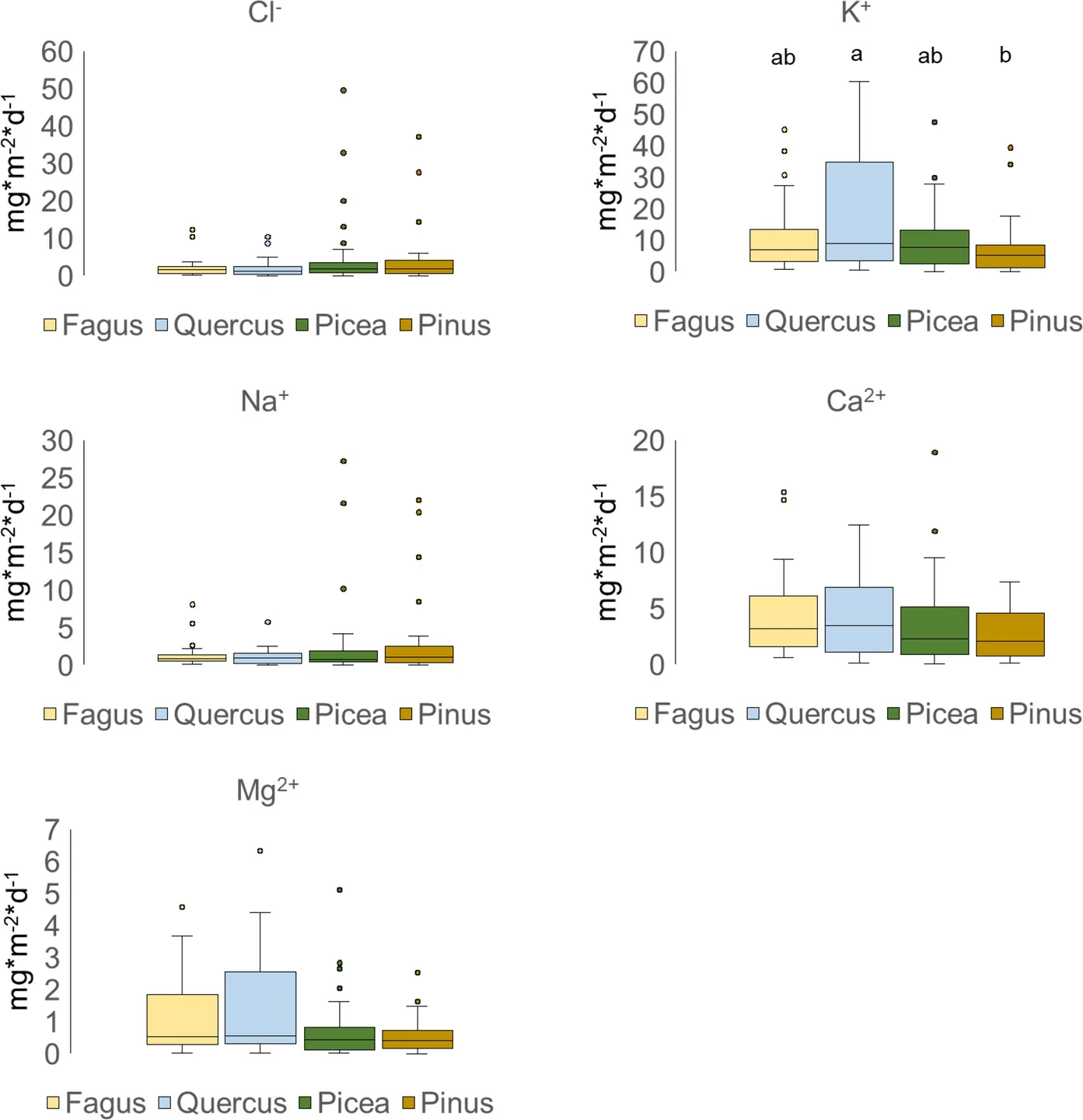
Throughfall compound fluxes measured in the period April–June 2018 (i.e., weeks 14–26) in *Fagus* (*n* = 27 TF samples), *Quercus* (*n* = 25 TF samples), *Picea* (*n* = 65 TF samples), and *Pinus* (*n* = 36 TF samples) stands. In the boxplots, the box represents the interquartile range (IQR; 25th to 75th percentiles), the inner line indicates the median, the whiskers delimit the minimum and maximum non-outlier values (i.e., 1.5 × IQR), and individual dots represent outliers. Different letters are reported for ions that displayed significant differences (*p* < 0.05) between the tree genera

Throughfall compound fluxes measured over the common period April–June 2018 on a total of *n* = 153 samples collected at the *Fagus*, *Quercus*, *Picea*, and *Pinus* plots, are represented in Fig. 6; mean (± SD) values are reported in Table S2.

Significant differences emerged between the tree genera for DON, PO_4_^3−^-P, and K^+^, with higher values in *Quercus* than in the other three genera (Fig. 6).

### DOC characteristics

**Fig. 7:**
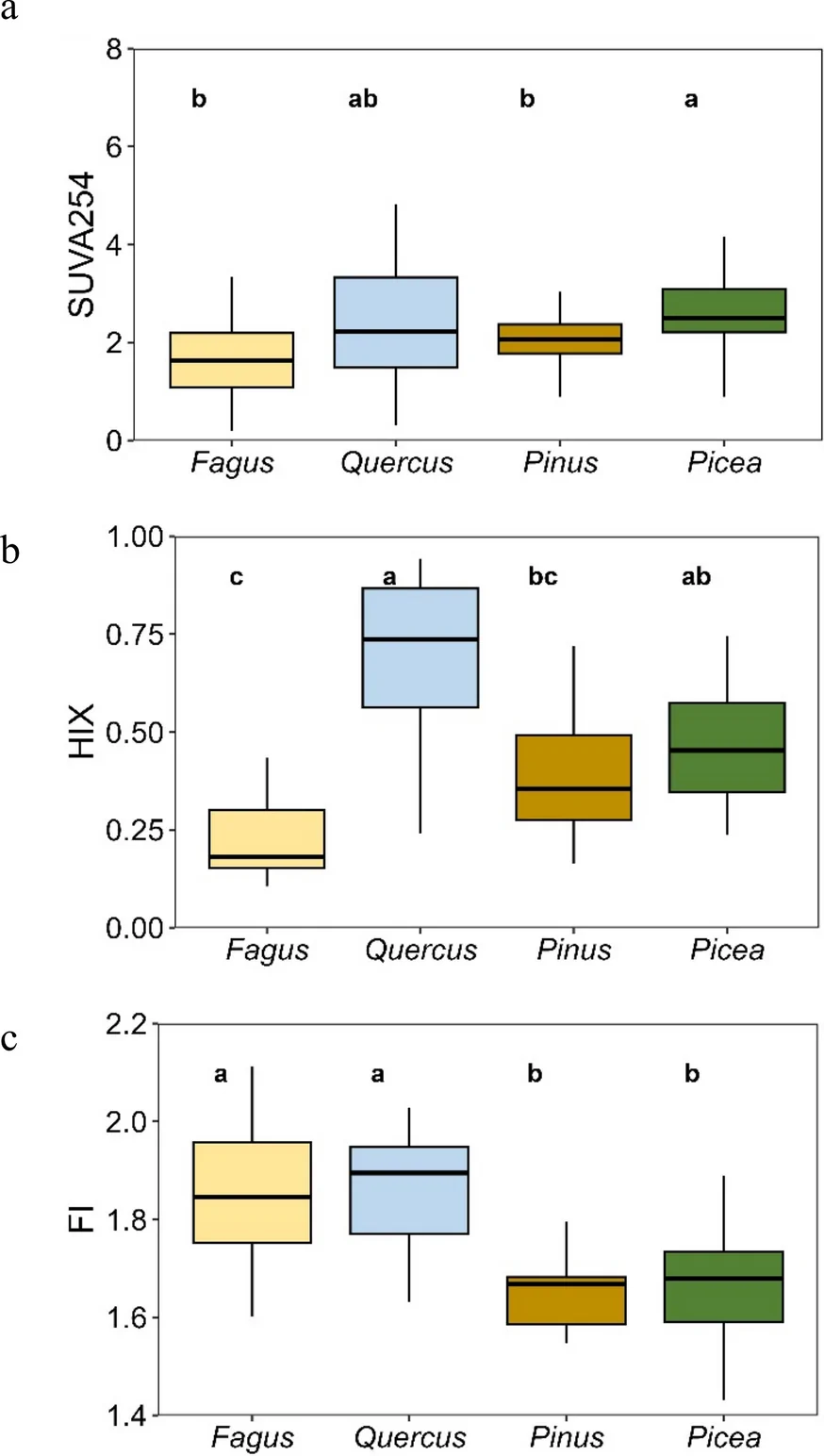
Dissolved organic carbon characteristics: **a** Specific UV Absorbance at 254 nm (SUVA_254_); **b** Humification Index (HIX); **c** Fluorescence Index (FI) for the four tree genera. Boxes show the distribution of data around the median (black line) for each genus. Letter symbols stand for significantly different groups

The biochemical stability (SUVA_254_), humification index (HIX) and fluorescence index (FI) of TF DOC for each tree genus are shown in Fig. 7.

The biochemical stability (SUVA_254_), proxy for the aromatic content of DOC, was significantly higher in *Picea* than in *Pinus* and in *Fagus* TF. The humification index (HIX), proxy for the degree of decomposition and transformation of organic matter, was significantly higher in *Quercus* TF compared with *Pinus* and *Fagus*; a significant difference was evidenced also between *Picea* and *Fagus*. Throughfall samples from *Fagus* and *Quercus* showed a significantly higher FI compared to those from *Pinus* and *Picea*, with median values around 1.9 (range between 1.6 and 2.1) for the broadleaf species, and around 1.7 (range of 1.4 and 2.05) for the conifer species. Most samples displayed FI values within 1.4 and 1.9 indicating a mix of origins, but likely with more microbial origin for the broadleaf pollen (Fig. 7).

### Relationships between pollen deposition and throughfall compound fluxes

Linear mixed-effects models revealed significant (*p* < 0.05) associations between pollen deposition rates and TF compound fluxes, although the strength of these relationships varied considerably across pollen taxa and chemical compounds (Table 1). Overall, all statistically significant associations identified by the LMMs were positive, suggesting that increased pollen deposition corresponded to elevated TF compound fluxes. The highest variance was explained by *Quercus* pollen for NH_4_^+^ (77.8%), and TDN (55.0%), while the lowest variance was explained by *Picea* pollen for TDN (6.1%). Potassium fluxes were significantly explained by *Fagus*, *Quercus*, *Pinus*, and *Picea* pollen deposition in the corresponding tree genus-dominated stands (on average, 33.2% ± 9.7%). When considering the combined input of *Pinus* and *Picea* pollen, significant positive associations were found for all the nutrient fluxes, excluded PO₄^3^⁻. *Quercus* and *Fagus* + *Quercus* pollen together evidenced the only weak negative associations with NO₃⁻ (*p* = 0.078 and *p* = 0.061, respectively).

**Table 1:** Results of mixed-effects models predicting throughfall fluxes (mg m^−2^ d^−1^) using pollen deposition rate (of the main tree genus or of all pollen taxa) (P m^−2 ^∙d^−1^) as a fixed effect. The estimate of the fixed effect (R^2^m; marginal *R*^2^) and its significance level are provided below each compound; ns: not significant, (*): *p* < 0.1, *: *p* < 0.05, **: *p* < 0.01, ***: *p* < 0.001

Tree genus	Weeks	Samples, n	Pollen taxon	NPOC	DON	TDN	NO_2_^−^-N	NO_3_^−^-N	NH_4_^+^-N	PO_4_^3−^	SO_4_^2−^	Cl^−^	K^+^	Na^+^	Ca^2+^	Mg^2+^
*Fagus*	14–22	16	*Fagus*	ns	ns	ns	ns	ns	ns	ns	ns	ns	0.141*	ns	ns	0.101(*)
			*R*^*2*^*m*										0.274			0.218
			total	0.346**	0.372*	0.196*	ns	ns	ns	ns	0.155(*)	ns	0.462**	ns	0.284*	0.377**
			*R*^*2*^*m*	0.419	0.339	0.365					0.242		0.417		0.371	0.513
*Quercus*	14–22	14	*Quercus*	0.093**	0.187*	0.183**	ns	−0.22(*)	0.389***	0.292*	ns	ns	0.188*	ns	ns	0.131(*)
			*R*^*2*^*m*	0.17	0.45	0.55		0.258	0.778	0.489			0.394			0.249
			total	0.579**	0.555*	0.544***	ns	ns	0.581**	0.949**	0.558**	0.519*	0.61**	0.536*	0.386*	0.641**
			*R*^*2*^*m*	0.687	0.506	0.544			0.140	0.667	0.486	0.337	0.538	0.312	0.334	0.617
*Fagus* + *Quercus*	14–22	30	*Fagus* + *Quercus*	0.086*	0.093*	0.08*	ns	−0.153(*)	0.164*	0.128(*)	ns	ns	0.175***	ns	ns	0.104**
			*R*^*2*^*m*	0.15	0.138	0.195		0.119	0.145	0.107			0.364			0.206
			total	0.444***	0.436***	0.325***	ns	ns	0.164*	0.517	0.262**	0.287*	0.572***	0.284*	0.305**	0.460***
			*R*^*2*^*m*	0.538	0.403	0.429			0.122	0.238	0.263	0.174	0.516	0.166	0.29	0.506
*Pinus*	16–25	24	*Pinus*	0.321**	0.372(*)	0.511***	0.48**	0.453*	0.334**	ns	0.297**	0.4**	0.361**	0.374**	ns	0.264*
			*R*^*2*^*m*	0.361	0.138	0.499	0.426	0.26	0.115		0.365	0.353	0.433	0.369		0.242
			total	0.459**	0.639*	0.623***	0.716***	0.556*	0.406**	ns	0.408**	0.598***	0.549***	0.541**	0.298*	0.43**
			*R*^*2*^*m*	0.402	0.222	0.499	0.517	0.211	0.087		0.379	0.442	0.548	0.427	0.209	0.35
*Picea*	14–23	40	*Picea*	ns	0.271**	0.169*	0.382**	ns	0.241**	0.256**	0.164*	ns	0.226**	ns	0.143(*)	0.133(*)
			*R*^*2*^*m*		0.195	0.061	0.264		0.065	0.218	0.135		0.226		0.099	0.094
			total	ns	ns	ns	0.241(*)	ns	0.143(*)	ns	ns	ns	ns	ns	ns	ns
			*R*^*2*^*m*				0.074		0.014							
*Pinus* + *Picea*	14–25	64	*Pinus* + *Picea*	0.199**	0.289**	0.229**	0.374***	0.274**	0.237***	ns	0.212***	0.203**	0.261***	0.178**	0.171**	0.172**
			*R*^*2*^*m*	0.138	0.125	0.091	0.21	0.092	0.05		0.188	0.105	0.226	0.089	0.115	0.119
			*total*	0.182*	0.293**	0.193*	0.382***	0.237*	0.218**	ns	0.182**	0.164*	0.234**	0.133(*)	0.162*	0.152*
			*R*^*2*^*m*	0.099	0.111	0.052	0.186	0.057	0.033		0.119	0.059	0.155	0.042	0.09	0.079

## Discussion

This study provides, for the first time, temporally resolved, taxon-level data on pollen deposition in TF, along with its correlations with TF chemical composition in European forests. The evaluation of the temporal alignment between the TF sampling periods and the flowering phenology of the six tree genera initially included in the study led to the exclusion of *Abies* and *Larix* stand data because most of the samples were collected outside the main pollen season. Thus, the study focused on *Fagus*, *Quercus*, *Picea*, and *Pinus* stand data.

In *Fagus*, *Picea,* and *Pinus* stands, the corresponding pollen dominated the TF pollen spectrum, while in *Quercus* stands it was second in abundance after *Pinus* pollen. *Pinus* produces huge numbers of pollen that can travel large distances via the wind due to the presence of the air sacs that increase its buoyancy (Szczepanek et al., 2017); pollen from nearby areas may supplement or even exceed locally sourced deposition, and this is likely the reason why *Pinus* pollen was overall largely predominant in our samples. These observations highlight the complex interplay between local vegetation, local-to-long distance pollen transport (Zemmer et al., 2024), and flowering phenology in shaping the pollen spectrum.

The evaluation of the time-course of pollen deposition amount in TF provided additional insight into the genus-specific flowering phenological phases and proved to be a useful reference to interpret the concurrent TF chemical composition patterns.

All the statistically significant relationships between the amounts of pollen grains deposited in TF and the compound fluxes were positive, suggesting that increased pollen content in TF corresponds to TF nutrient enrichment, with different proportions of explained variance. For instance, while *Pinus* pollen was significantly associated with several compounds (10 out of 13), the variance explained by the pollen itself ranged between 11.5% (for NH_4_^+^) and 50% (for TDN), indicating that pollen deposition can explain a variable portion of the variance in TF chemistry. These results underline that the overall role of pollen in nutrient flux variations can be relevant but that other factors, such as canopy structure, atmospheric dry deposition, or local meteorological conditions, should be considered in the models. The pollen amounts of all the four tree genera considered (*Fagus*, *Quercus*, *Picea*, *Pinus*) deposited in the TF samples were linked with the amount of K^+^. Since K^+^ is one of the predominant ions contained in pollen grains (Cerceau-Larrival et al., 1991; Kostić et al., 2015; Valverde et al., 2023), it is plausible that pollen, both washed away from flowers/crowns by precipitation or dry deposited, is a direct source of this ion in the TF during the main pollen season, as suggested earlier by Lindberg et al. (1986).

Although calcium is also one of the main constituents of pollen, its flux in TF showed limited or no link with pollen amount of the four tree genera, suggesting it could have been mostly in insoluble form or consumed, e.g., in the germination process, wherein it is a critical factor (Steinhorst et al., 2013). Broadleaved pollen (*Fagus* + *Quercus*) significantly explained NPOC, DON, TDN, NH₄^+^, and Mg^2+^ fluxes, while pollen from conifers (*Pinus* + *Picea*) was significantly linked with all the compounds, except PO_4_^3−^. These patterns may reflect differences in pollen chemistry among taxonomic groups (Kenđel & Zimmermann, 2020) or environmental interactions during deposition processes (Rösel et al., 2012). *Fagus* + *Quercus* pollen and *Quercus* pollen alone showed a weak negative link with NO_3_^−^. Similarly, a negative correlation was observed by Verstraeten et al. (2023) between NO_3_^−^ in TF and airborne pollen concentrations from 6 to 29 year-long time series for 22 *Fagus* stands across Europe. In our study, when considering the sum of all pollen taxa (including conifers) collected in the *Fagus* and *Quercus* stands, such a link was not present, suggesting that NO_3_^−^ reduction could take place predominantly in the presence of pollen of broadleaves. However, interpreting this negative relationship requires a cautious approach, as several non-mutually exclusive pathways could explain it. In vitro experiments have demonstrated that pollen and its associated microbiota can transform extracellular NO_3_^−​^ into NO_2_^−^​ and N_2_​O (Limić et al., 2025). This effect was particularly pronounced in broadleaved species such as *Fagus sylvatica* and *Quercus robur*, while it was significantly weaker or absent in coniferous species such as *Pinus sylvestris* and *Picea abies*. While these laboratory results provide a potential biological mechanism for the observed field patterns, the complexity of the forest canopy environment makes it difficult to confirm that these same processes occur in real field conditions. The negative link observed between broadleaved pollen deposition and NO_3_^−^ fluxes could also reflect lower atmospheric deposition during the specific period of *Fagus* and *Quercus* flowering, but to verify this mechanism, high-resolution data on atmospheric NO_x_ concentrations would be needed. Finally, NO_3_^−^ can be removed by tree canopies since they act as extremely efficient sinks for nitrogen interception and uptake (Klopatek et al., 2006; Guerrieri et al., 2024). This single-year field study cannot resolve the mechanisms mentioned; future research, utilizing e.g., stable isotope tracing or controlled field exclusion experiments, is necessary to identify the precise pathways of nitrate modulation during the pollen season.

Pollen was related to DOC flux as well as to its quality, which distinctly differed between the tree genera. Higher SUVA_254_ values indicate more aromatic, humic-like substances, which are generally more stable and less biodegradable. These were highest in *Picea* stands, in which needles in general tend to release more aromatic and humified compounds such as lignin derivatives and tannins, compared to *Pinus*. *Fagus* typically contributes more labile, aliphatic DOC, which is less aromatic and more biodegradable, as found in this study in comparison to *Quercus*, whose leaves — like those of *Picea* — are rich in polyphenols and tannins. The higher values of humification index in *Quercus* stands in comparison to *Picea*, *Pinus* and *Fagus*, reflect a higher level of decomposition and transformation of organic matter into humic substances. Although DOC in TF, as well as potassium, is considered to derive almost exclusively from vegetative sources (McDowell et al., 2020), the high values of fluorescence index (FI) for *Fagus* and *Quercus* suggest that, in these two stands, microbes may have played a relevant role in processing DOC. The lower FI values for the two conifers indicate a mix of plant and microbial origin for DOC.

In synthesis, the results of this study support our initial hypothesis that variations in throughfall chemistry in European temperate and boreal forests during the initial vegetative — and thus flowering — season are linked to pollen abundance. This is taxon-specific and particularly marked for some nutrients, e.g., potassium, total dissolved nitrogen and ammonia. Our findings suggest that including pollen deposition metrics in long-term forest monitoring could provide a more comprehensive picture of nutrient cycling, particularly in light of ongoing climate-driven changes in flowering phenology that are leading to an overall earlier onset of the pollen season and an increase in airborne pollen amounts of arboreal taxa (Bruffaerts et al., 2018; Cristofolini et al., 2024; Montiel et al., 2025).

Reproductive phenology, particularly masting behaviour in species such as *Fagus* and *Quercus* (Nussbaumer et al., 2016), introduces strong interannual variability in pollen inputs. In addition to interannual variability in pollen production within a single species, which may also be linked to the meteorological conditions (Nielsen et al., 2010), an interspecific variability in pollen influx must also be considered. Species-specific differences in pollen production, dispersal, and deposition shape this variation (Poska & Pidek, 2010). The morphology of pollen grains, specifically size and shape, affects their aerodynamic properties, with larger and heavier grains tending to settle closer to their source due to gravitational forces (Hofmann et al., 2014). Moreover, variations in pollen abundance among species may be explained by a trade-off between pollen grain size and amount produced (Vonhof & Harder, 1995), which likely reflects a resource allocation strategy.

The analysis of the TF chemical composition and pollen deposited in the same samples probably provides the most accurate information to verify the link between pollen amount and nutrient cycling, but it is quite a laborious method. One potential alternative approach could be the deployment of aerobiological samplers, either passive or active, adequately positioned beneath the forest canopy (as well as in open field) to simultaneously collect aerobiological samples to obtain data on pollen deposition rates alongside — but independently from — chemical deposition measurements.

This study is subject to certain limitations, notably the restricted one-year time span. Future research, based on multi-year observations, could include data on canopy architecture (Pypker et al., 2011), age of stand (Klopatek et al., 2006), phenology (Staelens et al., 2011), and insect infestations that alter canopy nutrient dynamics (Michalzik, 2011; Pitman et al., 2010), which may affect the strength of causal inference, to further disentangle the direct influence of pollen from other variables.

## Conclusions

By quantifying pollen deposition directly within the same TF samples analysed for chemistry, this study provides evidence that pollen is a measurable and taxon-specific contributor to nutrient fluxes. While positive links with ions like K⁺ underscore its role as a nutrient source, these findings from a single-year correlative study should be viewed as a baseline. The contribution of pollen to TF chemistry calls for a reconceptualization of its role in forest ecosystems; however, a full mechanistic understanding and an assessment of long-term dynamics will require further multi-year research to account for interannual variability such as masting and climate- and N deposition-driven fluctuations.

As climate change impacts reproductive phenology, incorporating pollen surveys into forest observation networks will be important for accurately assessing nutrient budgets and ecosystem responses.

## Supplementary Information

Below is the link to the electronic supplementary material.

Supplementary Material 1 (DOCX 1.01 MB)

## Data Availability

The throughfall data can be obtained by submitting an official data request through the ICP Forests website http://icp-forest.net/. The pollen dataset generated and analysed during the current study is available from the corresponding author on reasonable request.
