# Supplementary material for: Deposited pollen from dominant tree genera drives nutrient composition of throughfall in European forests
Source: Environ Monit Assess. 2026 Sep 9;198(10):1053. doi: 10.1007/s10661-026-15883-0 (PMC13558327; doi:10.1007/s10661-026-15883-0)
Supplement: Supplementary file 1 [file 10661_2026_15883_MOESM1_ESM.docx]

Table S1. List of Countries and ICP Forest plots with elevation, and number of throughfall samples for each tree genus analyzed for pollen content and chemical composition.

| Country | ICP Forests plot | Elevation (m a.s.l.) | *Abies* | *Fagus* | *Larix* | *Picea* | *Pinus* | *Quercus* |
| --- | --- | --- | --- | --- | --- | --- | --- | --- |
| Belgium | 11 | 31 |  | 5 |  |  |  |  |
|  | 14 | 35 |  |  |  |  | 5 |  |
|  | 15 | 14 |  |  |  |  | 5 |  |
|  | 16 | 26 |  |  |  |  |  | 5 |
|  | 21 | 129 |  | 5 |  |  |  |  |
| Finland | 1 | 125 |  |  |  |  | 3 |  |
|  | 3 | 275 |  |  |  | 3 |  |  |
|  | 12 | 125 |  |  |  | 3 |  |  |
|  | 13 | 125 |  |  |  |  | 3 |  |
|  | 19 | 175 |  |  |  | 3 |  |  |
|  | 20 | 175 |  |  |  |  | 2 |  |
| France | 6 | 149 |  |  |  |  |  | 2 |
|  | 17 | 127 |  |  |  |  |  | 2 |
|  | 30 | 80 |  |  |  |  |  | 1 |
|  | 37 | 480 |  |  |  | 1 |  |  |
|  | 46 | 650 |  |  |  | 1 |  |  |
|  | 57 | 1400 |  | 2 |  |  |  |  |
|  | 63 | 400 |  | 2 |  |  |  |  |
|  | 84 | 175 |  |  |  |  | 2 |  |
|  | 90 | 1360 | 2 |  |  |  |  |  |
|  | 93 | 950 | 2 |  |  |  |  |  |
|  | 96 | 1100 | 2 |  |  |  |  |  |
|  | 98 | 400 | 2 |  |  |  |  |  |
|  | 100 | 680 | 2 |  |  |  |  |  |
| Germany | 901 | 425 |  |  |  |  | 5 |  |
|  | 902 | 425 |  |  |  | 5 |  |  |
|  | 903 | 825 |  | 6 |  |  |  |  |
|  | 904 | 1475 |  |  | 8 |  |  |  |
|  | 905 | 475 |  |  |  |  | 6 |  |
|  | 906 | 525 |  |  |  | 7 |  |  |
|  | 907 | 425 |  | 4 |  |  |  |  |
|  | 908 | 825 |  |  |  | 6 |  |  |
|  | 923 | 525 |  |  |  | 6 |  |  |
|  | 922 | 1075 |  |  |  | 6 |  |  |
|  | 913 | 475 |  |  |  |  |  | 6 |
|  | 920 | 675 |  |  |  | 6 |  |  |
|  | 914 | 475 |  |  |  |  |  | 3 |
|  | 921 | 325 |  |  |  |  |  | 5 |
| Italy | 17 | 1800 |  |  |  | 11 |  |  |
| Norway | 1 | 198 |  |  |  | 4 |  |  |
|  | 19 | 275 |  |  |  | 3 |  |  |
|  | 7 | 560 |  |  |  | 4 |  |  |
| Switzerland | 2 | 1520 |  |  |  | 2 |  |  |
|  | 3 | 1130 |  | 2 |  |  |  |  |
|  | 4 | 1870 |  |  |  |  | 1 |  |
|  | 5 | 1370 |  |  |  | 1 |  |  |
|  | 18 | 1640 |  |  |  | 1 |  |  |
|  | 7 | 680 |  |  |  |  |  | 1 |
|  | 19 | 500 |  | 1 |  |  |  |  |
|  | 8 | 810 |  | 2 |  |  |  |  |
|  | 10 | 1900 |  |  |  |  | 1 |  |
|  | 12 | 950 |  |  |  |  |  | 2 |
|  | 13 | 490 |  | 1 |  |  |  |  |
|  | 16 | 730 |  | 1 |  |  |  |  |
|  | 14 | 680 |  |  |  |  | 1 |  |
|  | 15 | 480 | 1 |  |  |  |  |  |
| United Kingdom | 512 | 75 |  |  |  |  |  | 3 |
|  | 715 | 25 |  |  |  |  | 3 |  |
|  | 718 | 125 |  |  |  |  | 3 |  |
|  | 919 | 275 |  |  |  | 3 |  |  |
| TOT plot | 60 |  | 6 | 11 | 1 | 19 | 13 | 10 |
| TOT samples |  |  | 11 | 31 | 8 | 76 | 40 | 30 |

Table S2. Mean (± sd) daily values of throughfall element fluxes (mg∙m^-2^∙d^-1^) for each tree genus calculated for the period April-June 2018. NPOC = non-purgeable organic carbon, DON = dissolved organic nitrogen, TDN = total dissolved nitrogen.

| Tree genus | NPOC |  | sd | DON |  |  | TDN |  |  | NO_2_^-^-N |  | sd | NO_3_^-^-N |  | sd | NH_4_^+^-N |  | sd | PO_4_^3-^P |  | sd | SO_4_^2-^ |  | sd | Cl^-^ |  | sd | K^+^ |  | sd | Na^+^ |  | sd | Ca^2+^ |  | sd | Mg^+^ |  | sd |
| --- | --- | --- | --- | --- | --- | --- | --- | --- | --- | --- | --- | --- | --- | --- | --- | --- | --- | --- | --- | --- | --- | --- | --- | --- | --- | --- | --- | --- | --- | --- | --- | --- | --- | --- | --- | --- | --- | --- | --- |
| Fagus (n=27) | 25.0 | ± | 19.20 | 3.9 | ± | 3.23 | 8.2 | ± | 5.08 | 0.11 | ± | 0.275 | 1.9 | ± | 1.70 | 2.2 | ± | 3.21 | 0.75 | ± | 1.788 | 3.3 | ± | 2.24 | 2.2 | ± | 2.83 | 12.3 | ± | 12.41 | 1.4 | ± | 1.71 | 4.6 | ± | 3.95 | 1.20 | ± | 1.323 |
| Quercus (n=25) | 44.3 | ± | 44.54 | 5.6 | ± | 5.86 | 11.8 | ± | 11.00 | 0.14 | ± | 0.391 | 1.1 | ± | 1.41 | 5.0 | ± | 6.19 | 3.28 | ± | 4.764 | 4.4 | ± | 3.95 | 2.2 | ± | 2.64 | 19.0 | ± | 19.35 | 1.4 | ± | 1.55 | 4.2 | ± | 3.68 | 1.44 | ± | 1.590 |
| Picea (n=65) | 38.4 | ± | 45.84 | 3.3 | ± | 3.39 | 8.3 | ± | 7.95 | 0.21 | ± | 0.480 | 2.4 | ± | 2.75 | 2.4 | ± | 3.21 | 0.52 | ± | 1.299 | 3.4 | ± | 2.75 | 4.0 | ± | 7.57 | 9.5 | ± | 8.99 | 2.0 | ± | 4.31 | 3.3 | ± | 3.33 | 0.66 | ± | 0.831 |
| Pinus (n=36) | 35.5 | ± | 33.80 | 2.8 | ± | 4.46 | 9.5 | ± | 11.09 | 0.12 | ± | 0.334 | 2.9 | ± | 3.85 | 3.7 | ± | 4.34 | 0.27 | ± | 0.791 | 3.5 | ± | 3.41 | 4.9 | ± | 9.37 | 7.1 | ± | 8.60 | 2.9 | ± | 5.24 | 2.7 | ± | 2.11 | 0.59 | ± | 0.594 |

Figure S1. Timing and length of the 196 throughfall samples collected in 2018 in 60 ICP Forests Level II plots across Europe, grouped for tree genus and, within each genus, listed per country and plot ID; each row represents a single sample.

Figure S2. Pollen grains belonging to the main tree genus characterizing the forest stands where throughfall samples were collected in 2018 in 60 ICP Forests Level II plots across Europe.
